# MetaTM - a consensus method for transmembrane protein topology prediction

**DOI:** 10.1186/1471-2105-10-314

**Published:** 2009-09-28

**Authors:** Martin Klammer, David N Messina, Thomas Schmitt, Erik LL Sonnhammer

**Affiliations:** 1 Stockholm Bioinformatics Centre, Albanova, Stockholm University, 10691 Stockholm, Sweden

## Abstract

**Background:**

Transmembrane (TM) proteins are proteins that span a biological membrane one or more times. As their 3-D structures are hard to determine, experiments focus on identifying their topology (i. e. which parts of the amino acid sequence are buried in the membrane and which are located on either side of the membrane), but only a few topologies are known. Consequently, various computational TM topology predictors have been developed, but their accuracies are far from perfect. The prediction quality can be improved by applying a consensus approach, which combines results of several predictors to yield a more reliable result.

**Results:**

A novel TM consensus method, named MetaTM, is proposed in this work. MetaTM is based on support vector machine models and combines the results of six TM topology predictors and two signal peptide predictors. On a large data set comprising 1460 sequences of TM proteins with known topologies and 2362 globular protein sequences it correctly predicts 86.7% of all topologies.

**Conclusion:**

Combining several TM predictors in a consensus prediction framework improves overall accuracy compared to any of the individual methods. Our proposed SVM-based system also has higher accuracy than a previous consensus predictor. MetaTM is made available both as downloadable source code and as DAS server at

## Background

Transmembrane proteins are proteins that span the biological membrane one or more times. An estimated 20 - 30% of all genes in an organism code for TM proteins [1,2]. Virtually all communication and transportation between the inside and the outside of a cell is mediated by them. Furthermore they are vital for cell recognition and cell adhesion and serve as receptors. This makes them especially interesting for medicine, since almost half of all present-day drug targets are TM proteins [3].

Two major types of TM proteins can be distinguished: α-helical TM proteins and TM β-barrels. Proteins of the α-helical class are by far the more abundant, therefore only this class will be considered in this paper. Although TM proteins make up about a fifth of all known protein sequences, only about one single percent of all known 3-D structures are TM proteins [4]. This is due to the fact that transmembrane proteins are very hard to crystallize. There are methods to determine the rough membrane-spanning topology of TM proteins (e. g. reporter fusion, site tagging, antibodies or mass spectrometry), but even this has only been done for less than a thousandth of all known protein sequences. To bridge this gap, there is a great need for computational prediction.

The topology of an α-helical TM protein describes which parts of the amino-acid sequence are buried in the lipid bilayer, and which are facing the aqueous environment on either side of the cell (i. e. the cytoplasmic or the non-cytoplasmic side). The portions that lie within the bilayer are termed TM segments, while the ones on either the in- or the outside of the cell (or organelle) are mostly called loops. Since loops alternate between the inside and the outside of the membrane, the topology information can be reduced to the location of the first loop (N-terminal location) and the position of all TM segments.

An α-helical TM segment consists of an approximately 15 - 30 residues long region with an over-representation of hydrophobic residues [5]. This fact makes the computational prediction of TM proteins a rewarding task. However, there are also other parts of proteins that have the same physico-chemical properties, e. g. the core region of signal peptides, which are short pro-peptides (i. e. cleaved off) that guide the membrane translocation of mature proteins. Their appearance often confuses TM topology predictors [5].

### *In silico *TM topology prediction

As the topology of a TM protein mostly depends on its primary amino-acid sequence, the computational prediction can be carried out fairly easily. A large number of TM topology predictors are available today, ranging from simple hydrophobicity analysis (e. g. TopPred [6]) to more complex methods based on hidden Markov models (e. g. TMHMM [7], HMMTOP [8], Phobius [5]) or artificial neural networks (e. g. PHDhtm [9], Memsat [10]).

The use of homologous sequences can improve the accuracy of TM topology predictors by up to 10% [11], thus many predictors support this kind of information (e. g. PHDhtm, HMMTOP, Memsat, Phobius in its homology-supporting version PolyPhobius [12]).

Current TM topology predictors are claimed to predict the correct topology for 70 - 85% of all proteins, but studies on whole-genome data show that this is an overestimation [13,14]. Furthermore, different predictors have different strengths and weaknesses. Some tend to over-predict TM segments, others are very conservative and miss more of them. Most predictors also tend to falsely predict signal peptides (SPs) as TM segments, whereas only a few of them can handle this problem (e. g. Phobius, Memsat).

Due to these different strengths and weaknesses of several predictors, it seems natural to make attempts to combine them and make a prediction on a meta-level. This is called a consensus prediction. The aim is to reduce method-specific weaknesses and therefore yield higher accuracies. This can be achieved by building a predictor that combines the results of various methods and — by applying some weighting and heuristics — calculates a meta-result. This meta-result, which represents the consensus of all methods, is potentially more reliable than a single prediction [15]. Previous approaches to combining results into a consensus prediction include simple majority voting [16,17] and Bayesian Belief Networks [18].

### Incorporated Predictors

MetaTM uses six TM topology predictors to achieve a consensus prediction. These predictors are TopPred, PHDhtm, HMMTOP, TMHMM, PolyPhobius and Memsat. Four of these (PolyPhobius, PHDhtm, HMMTOP and Memsat) support the use of homology information (see also Table 1 for details). For PolyPhobius, PHDhtm and HMMTOP homologs are found via BLAST [19] searches against a protein sequence database. Subsequently, a multiple alignment is created with Kalign [20] and used as input for PolyPhobius and PHDhtm. HMMTOP does not require a multiple alignment; it can handle the multi-FASTA file resulting from the BLAST search directly. Memsat performs a PSI-BLAST [21] search and uses the resulting sequence profile as input for the predictor.

**Table 1:** TM topology predictors

**Method**	**Version**	**TM pred.**	**SP pred.**	**Homology**
TopPred	1.0	Yes	No	No
PHDhtm	2.1	Yes	No	Yes
HMMTOP	2.1	Yes	No	Yes
TMHMM	2.0	Yes	No	No
PolyPhobius	1.0	Yes	Yes	Yes
Memsat	3.0	Yes	Yes	Yes
SignalP	3.0	No	Yes	No

Features of the six TM topology predictors incorporated in MetaTM, plus the SP-only predictor SignalP.

Another important feature is the SP prediction to avoid mutual false-classifications of TM segments and SPs. Two TM topology predictors (PolyPhobius and Memsat) are also capable of predicting signal peptides. However, Memsat does not deliver very reliable results for signal peptides [10] and therefore was not used in the consensus predictor for this kind of prediction. Additionally, SignalP [22], a method that only predicts SPs, was included, too. This method can be thought of as an assisting predictor to reach a consensus on the SP prediction together with PolyPhobius.

### Support Vector Machines

In order to build a model that predicts TM topology from a set of inputs, one needs to employ a machine learning method. An increasingly popular technique is the support vector machine (SVM) [23]. Here, non-linear dependencies between the input features are handled by mapping the input to a higher dimensional feature space by means of a kernel function. In kernel feature space, the SVM will construct a hyperplane that separates the two data sets with a maximal margin. This delivers good generalization and the ability to capture non-linear behavior. SVMs have been used successfully for a large number of bioinformatics prediction tasks [24].

## Results

### The MetaTM algorithm

On the top level the consensus prediction is split into two major parts: (1) the segments consensus for finding TM segments and signal peptides (SPs), and (2) the N-terminal consensus. The latter determines whether the N-terminal end of the amino-acid sequence is located on the cytoplasmic or non-cytoplasmic side (also referred to as inside and outside, respectively) of the membrane. They are both predicted independently based on two different SVM models and afterwards combined into a final consensus topology.

#### Segments consensus

**Figure 1:**
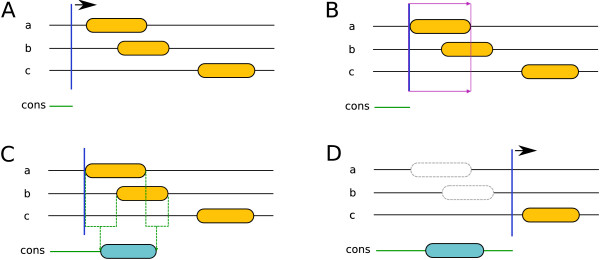
**Segments consensus workflow**. For clarity only three predictors are drawn. The orange elements represent predicted TM segments. (A) Scanning the results for the first segment. (B) Detecting overlapping segments and voting whether the group of overlapping segments should be added to the consensus result. (C) If the voting was positive (i. e. the SVM model predicts a TM segment - we assume that this is true in this case), a segment with averaged start and end positions was added to the consensus result (blue segment). (D) Masking the used segments and scanning for the next one.

The segments consensus can be roughly subdivided into the following steps: initially, the method scans the result of all incorporated predictors towards the C-terminus for the first occurring segment (see Figure 1A). If such a segment is found, segments from the other predictors that overlap with the first one are detected. This can also be thought of as applying a window reaching from the beginning of the first segment to its end, and then looking for other segments that intersect with this window (see Figure 1B). Subsequently, the SVM segment model predicts the consensus, which can be either a TM segment, an SP or no segment (i. e. loop). We termed this procedure *voting*.

To have the consensus predicted by the SVM segment model, the results of the incorporated predictors for each window have to be encoded as a vector. In this case they are represented by a nine-dimensional vector with the following boolean values: Six for the TM topology predictors, two for the SP predictors, and finally one that indicates if the current window is the first for the current query sequence. This last value is an additional indicator for the prediction of signal peptides, as they can only appear at the N-terminal end of a sequence (and therefore only within the first window of a query sequence).

If the voting was positive (i. e. either an SP or TM segment should be added to the consensus prediction), the averages of the overlapping segments' start and end positions are calculated, respectively. If a window contains SPs and TM segments, only those segments which are of the same class as that predicted by the SVM model are used for the averaging (see also Figure 1C). Then all segments used for the prediction of the consensus segment are masked to not be used for following predictions. Afterwards the rest of the sequence is scanned for the next segment (see Figure 1D). Next, the cycle starts again from the beginning until no more unmasked segments are present.

**Figure 2:**
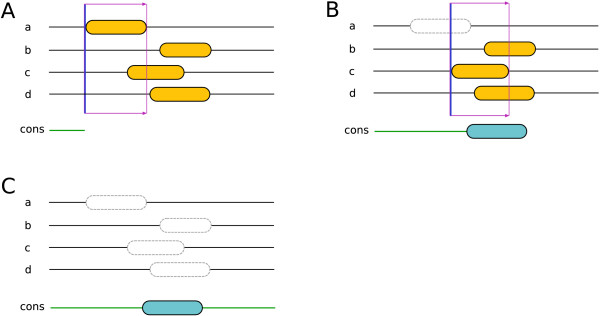
**The masking procedure**. For clarity only four predictors are drawn. The orange elements represent predicted TM segments. It is assumed in this example that three overlapping TM segments need to intersect with the voting window to have a positive consensus voting (i. e. the SVM model predicts a TM segment). (A) Only two segments are in the voting window, thus the voting result is negative. (B) Due to the negative voting result, the first segment in the window is masked and not used for further prediction anymore. The newly applied window contains three overlapping segments. (C) The consensus is reached in favor of the TM segment and therefore all overlapping segments are masked. Note that if both segments overlapping with the first window (the one displayed in (A)) had been excluded, there would not be any resulting consensus TM segments.

As shown in Figure 2, if the voting result for a given group of overlapping segments is negative (i. e. the SVM model predicts a loop), only the first segment will be masked and excluded from the further prediction process (see also Figure 2A/B). Only if the voting result is positive are all overlapping segments masked (see also Figure 2C). This increases the chance of detecting consensus segments.

#### N-terminal location consensus

The N-terminal consensus in MetaTM is reached by a voting mechanism based on a second SVM model. Each predictor contributes to the result by voting either for N-terminus on the inside (cytoplasmic side) or N-terminus on the outside (non-cytoplasmic side). The results are encoded as an eight-dimensional vector with the following boolean values: six for the TM topology predictors, where 0 stands for the N-terminus being located on the inside and 1 for the outside, and two for the SP prediction of PolyPhobius and SignalP, respectively (1 if an SP has been predicted, otherwise 0). The last two values assist the N-terminal prediction such that the occurrence of an SP automatically leads to an outside N-terminal location. This is due to the biological fact that SPs are cleaved off from the remainder of the protein after it has been inserted across the membrane.

### Comparison with single predictors

The prediction accuracy of MetaTM was assessed based on a data set containing 1460 TM protein sequences with known topologies and 2362 globular proteins, both with and without SPs [see Additional file 1]. This is the largest data set used for benchmarking TM topology predictors so far. To uncover strengths and weaknesses of MetaTM and other predictors, the data set was split into six categories (see Table 2).

**Table 2:** Data set categories

**Category**	**# Sequences**
TMsingleAndSP	282
TMmultiAndSP	63
TMsingleNoSP	237
TMmultiNoSP	878
GLBandSP	1275
GLBnoSP	1087

The six categories of the data set.

First, the quality of the N-terminal location prediction was assessed on the four categories that contain TM protein sequences (see Table 3). One can clearly see that PolyPhobius was the best single method when it comes to the prediction of sequences with signal peptides, as a predicted SP automatically leads to an N-terminus located on the outside. On the other hand, Memsat was the superior method for sequences without SPs. Although MetaTM was able to reach almost the same accuracy as PolyPhobius for the first category and matched PolyPhobius in the second category, its prediction quality is slightly less accurate than Memsat's for the latter two. However, since both PolyPhobius and Memsat predict rather poorly on two of the four sets, the overall performance of our consensus method was 5.6 and 8.3 percentage points better, respectively. Although it might not be obvious at first, all predictors contribute positively to the MetaTM result to some extent. Even TopPred, the weakest method, was able to tip the scales in favor of the correct prediction from time to time.

**Table 3:** N-terminal location prediction results

	**MT**	**PP**	**TH**	**HT**	**PH**	**MS**	**TP**
TMsingleAndSP	97.9%	**98.2%**	85.5%	67.4%	80.5%	81.9%	19.2%
TMmultiAndSP	**100.0%**	**100.0%**	73.0%	65.1%	66.7%	81.0%	30.2%
TMsingleNoSP	84.8%	69.2%	72.2%	79.8%	78.9%	**85.2%**	74.3%
TMmultiNoSP	86.7%	79.5%	78.1%	86.2%	72.6%	**87.8%**	71.8%
Average	**92.3%**	86.7%	77.2%	74.6%	74.7%	84.0%	48.8%
Average Rank	**1.75**	3.3	4.5	4.5	5.0	2.3	6.5

MT: MetaTM, PP: PolyPhobius, TH: TMHMM, HT: HMMTOP, PH: PHDhtm, MS: Memsat, TP: TopPred. Average Rank is the average of the ranks achieved by each predictor in each category.

The next comparison was done for the prediction of the correct number of TM segments on all six categories (see Table 4). Again, PolyPhobius delivered very good results for sequences with SPs involved, but MetaTM was even better than PolyPhobius in two of the three SP data sets and equally good in the third one. For TM proteins without preceding signal peptides, Memsat and HMMTOP are the best among the single predictors. Also in these categories, MetaTM performs very well and is best together with HMMTOP in TMsingleNoSP and only slightly behind HMMTOP in TMmultiNoSP. For sequences with neither SPs nor TM segments (i. e. those in GLBnoSP) TMHMM is able to reach the highest prediction accuracy, and our consensus method the second best. On average, MetaTM performed better than all single predictors, followed by PolyPhobius (1.9 percentage points less accurate) and Memsat (12.0 percentage points less accurate).

**Table 4:** Number of TM segments prediction results

	**MT**	**PP**	**TH**	**HT**	**PH**	**MS**	**TP**
TMsingleAndSP	**97.5%**	94.0%	79.8%	50.7%	82.6%	76.6%	9.9%
TMmultiAndSP	**90.5%**	88.9%	63.5%	63.5%	61.9%	71.4%	15.9%
TMsingleNoSP	**89.9%**	87.3%	87.8%	**89.9%**	85.7%	89.5%	72.2%
TMmultiNoSP	72.0%	70.6%	62.0%	**74.3%**	54.1%	72.0%	45.8%
GLBandSP	**94.9%**	93.4%	74.4%	37.5%	64.6%	69.7%	2.4%
GLBnoSP	97.0%	96.0%	**98.8%**	86.6%	71.6%	90.4%	49.5%
Average	**90.3%**	88.4%	77.7%	67.1%	70.1%	78.3%	32.6%
Average Rank	**1.3**	3.0	3.5	3.8	5.3	3.5	7.0

The abbreviations are the same as described in Table 3.

The prediction of the entire TM topology (i. e. the N-terminal location and TM segments, where each predicted TM segment has to overlap the experimentally determined one with at least 5 residues) can be considered the supreme discipline in TM topology prediction (see Table 5). The results look pretty much like a combination of the N-terminal location comparison and the TM segment number comparison. Especially in this — the most important — test, the performance of MetaTM was remarkably good. It was the best method in four of the six categories and in the remaining two sets MetaTM reached second place. On average the consensus method was 4.4 percentage points better than PolyPhobius, which took the second place, and 12.6 percentage points more accurate then Memsat, which was third in this comparison.

**Table 5:** Entire topology prediction results

	**MT**	**PP**	**TH**	**HT**	**PH**	**MS**	**TP**
TMsingleAndSP	**97.2%**	94.0%	75.5%	47.5%	79.1%	72.7%	7.1%
TMmultiAndSP	**84.1%**	81.0%	52.4%	57.1%	47.6%	65.1%	3.2%
TMsingleNoSP	**81.0%**	65.8%	68.8%	73.4%	69.6%	79.3%	53.6%
TMmultiNoSP	66.1%	63.7%	51.4%	65.7%	45.7%	**67.4%**	37.2%
GLBandSP	**94.9%**	93.4%	74.4%	37.5%	64.6%	69.7%	2.4%
GLBnoSP	97.0%	96.0%	**98.8%**	86.6%	71.6%	90.4%	49.5%
Average	**86.7%**	82.3%	70.2%	61.3%	63.0%	74.1%	25.5%
Average Rank	**1.3**	3.2	3.8	4.5	5.0	3.2	7.0

The abbreviations are the same as described in Table 3. The prediction for globular proteins was counted as correct if no TM segment had been predicted, regardless of the N-terminal location prediction.

All discussed comparisons so far have not directly involved the SP prediction. The reason why the SP comparison has not been considered so far is simply that most of the methods do not support their prediction. However, it is possible to assess the signal peptide prediction accuracy for PolyPhobius, SignalP and MetaTM. In Table 6 the prediction behavior of these three methods is plotted. While SignalP misses fewer signal peptides than PolyPhobius and MetaTM, it also over-predicts more (4.5 percentage points less accurate than MetaTM on average). MetaTM and PolyPhobius deliver quite similar results, although our consensus method is slightly better (1.2 percentage points on average).

**Table 6:** Signal peptide prediction results

	**MetaTM**	**PolyPhobius**	**SignalP**
missed	3.4%	5.1%	**2.2%**
over-predicted	**6.0%**	6.7%	16.3%
average error	**4.7%**	5.9%	9.2%

The error rate of the signal peptide prediction.

### Comparison with previous consensus predictors

We wanted to compare MetaTM's results with ConPred II [25], the most sophisticated of the existing consensus predictors. Unfortunately, the program is not available for local use, and an evaluation via its web interface was not feasible. Due to these limitations, a comparison between the two consensus methods could only be carried out by comparing MetaTM's results on the data set described in the ConPred II paper [25] with ConPred's results reported in the same. It has to be mentioned that this data set is rather small (231 sequences) and it only contains TM proteins without signal peptides. Thus, this comparison is far from complete. As one can see in Table 7, MetaTM and ConPred perform similarly on the N-terminal location prediction and the number of correctly predicted TM segments, although MetaTM achieved a slightly higher accuracy (1.8 and 0.5 percentage points better, respectively). However, when predicting the entire topology MetaTM was 2.6 percentage points better than ConPred. While MetaTM was always better than any single predictor, ConPred performed slightly worse than PolyPhobius in the case of entire topology prediction.

**Table 7:** ConPred II data set prediction results

	**MT**	**CP**	**PP**	**TH**	**HT**	**PH**	**MS**	**TP**
N-terminus	**84.9%**	83.1%	78.4%	73.2%	74.5%	66.7%	77.5%	68.8%
# Segments	**77.1%**	76.6%	74.0%	59.3%	66.7%	60.2%	71.4%	50.2%
Topology	**65.4%**	62.8%	63.2%	47.6%	51.9%	47.2%	58.4%	35.9%

The abbreviations are the same as described in Table 3, except CP: ConPred II.

## Discussion

The prediction of segment and N-terminal consensus is achieved by two different support vector machine (SVM) models. We also tried a different approach, where weights were assigned to the incorporated predictors based on their prediction quality. The idea was that methods which deliver more reliable results should contribute more to the consensus. The weights of all predictors voting for a certain state (e g. N-terminus inside or N-terminus outside in the case of N-terminal location prediction) were summed up and compared to each other. Subsequently, the state with the higher vote was considered to be the consensus result. This approach, although fairly simple, also delivered good results and was only about 1 percentage point less reliable than the approach using SVM.

As the prediction quality of MetaTM strongly depends on the results of the underlying predictors, the performance could be further improved by adding better methods or replacing poorly performing ones with them. Of course, our aim was to use only well-performing predictors, but new methods can easily be incorporated in the future.

## Conclusion

We have presented a novel TM consensus method, MetaTM, that predicts the transmembrane topology and signal peptides based on the results of seven single predictors. Although MetaTM was not able to deliver the best results in all data categories, it is the most reliable method on average in all three tests (i. e. N-terminal location, number of TM segments, entire topology). For predicting the entire topology of protein sequences, the most important test in TM topology prediction, MetaTM reached an average accuracy of 86.3%, which was 4.0 percentage points better than the result of the best single predictor. Furthermore, its average signal peptide prediction quality is also better than those of its incorporated SP predictors.

Compared to ConPred II, an existing consensus predictor, MetaTM was 2.6 percentage points more accurate in terms of entire topology prediction. Due to availability limitations of the ConPred II program, the prediction quality could only be compared based on the data set and results described in the ConPred II paper. Presumably, the results would have been even more clearly in favor of MetaTM if sequences with signal peptides had been in the ConPred data set, as ConPred II does not include SP predictors.

## Methods

### Data sets

The data set for the comparison with the single predictors comprises data from the recently published TOPDB database [26] and the data set that was originally compiled for Phobius [5]. TOPDB (revision 1) currently comprises 1452 α-helical TM protein sequences, of which 94 were excluded as they contain propeptides or membrane loops. The remaining 1358 sequences were combined with all 292 α-helical TM protein sequences and 2362 globular ones from the Phobius data set. There was some overlap of sequences between the two data sets, so duplicate entries were removed. This led to a final data set with 1460 TM protein sequences and 2362 globular ones, or 3822 sequences in total.

A disproportionate number of strong homologs in the selected data set could affect the result of the predictor comparison, as it could favor or disfavor a particular predictor. To rule out such bias, a pairwise comparison of all proteins in the data set was done. Only 70 pairs between 117 different proteins with more than 90% identity were found, ruling out an effect on the results.

The data set for the comparison with ConPred II comprising 231 α-helical TM protein sequences was downloaded from the predictor's homepage (see [25]). The performance of MetaTM was assessed with the underlying SVM models trained on the data set mentioned in the paragraph above. The results of ConPred II were taken from its paper [25] where the results are separately described for pro- and eukaryotic sequences. In our comparison, we did not make this distinction, so we recalculated the fractions of correct predictions for the entire set based on their reported results.

### Homology detection and MSA

In order to reduce the duration of time-consuming homology searching, a sub-database of UniProt/SwissProt (release 55.2) was created (called SwissMemProts). The idea for this sub-database was to extract all membrane proteins from SwissProt and use the resulting subset as the database for the homology search. Membrane proteins were detected by searching for the occurrence of the string membrane in the CC-section of each entry. This CC-block stores the annotation of the sequence (e. g. function, sub-cellular location). If the string was found in this section, the corresponding sequence was added to the sub-database. This filtering procedure reduced the number of entries from 362,782 to 75,083 without decreasing the accuracy of MetaTM's results.

Homologs for PolyPhobius, HMMTOP and PHDhtm were found with the BLAST algorithm [19] (blastall Version 2.2.16). The following parameters were set: -p blastp, -e 1e-5 and -b 50. Resulting homologous sequences were aligned with the Kalign 2.0 [20] multiple sequence alignment (MSA) tool using the default parameters, and the produced multiple alignment was passed to PolyPhobius and PHDhtm. HMMTOP does not require an aligned sequence; it rather takes the list of homologous sequences directly. The homology detection for Memsat was done with the default script that comes with the program and PSI-BLAST [21] (blastpgp Version 2.2.16).

### SVM models

The models used in the SVM voting mechanism were created with the libsvm [27] package (version 2.86). Two types of models have been designed, one for the segments consensus and one for the N-terminal location consensus. 10-fold cross validation was applied to train and test the model [see Additional file 2]. The cross validation sets were selected such that no proteins had more than 50% sequence identity matches between sets. The SVM models were produced with a Python script that comes with the package (called easy.py), using the radial basis function (RBF) kernel. This script automatically determines the optimal cost and RBF kernel parameters for each model, which is created during the cross validation process. For the final models (those that were trained on the entire data set), the optimized parameters are *C *= 2048 and γ = 4.88·10^-4 ^for the N-terminal location model, and *C *= 2 and γ = 0.125 for the segments model.

## Implementation

The program is written in Java 5.0 using the Eclipse framework. Additionally, a couple of C shell and Perl scripts were taken and modified from the SFINX meta server [28] in order to perform a conversion of the incorporated predictors' outputs into a modified version of the SFS format [28].

## Availability

MetaTM is made available both as downloadable source code and via DAS (distributed annotation system) [29] at . DAS allows the integration of MetaTM's results on an as-needed basis by special client-side software (e. g. DASher [30]).

## Authors' contributions

MK designed and implemented the application, and drafted the manuscript. DNM set up the DAS server. TS participated in designing the SVM models. ELLS oversaw the project and revised the draft manuscript. All authors have read and approved the final version of the manuscript.

## Supplementary Material

Additional file 1 **Predictor results**. The results of MetaTM and the other involved methods for each protein sequence in the data set. Click here for file

Additional file 2 **Cross validation sets**. The ten data sets used for cross validation of MetaTM. Click here for file

## References

[B1] Wallin E, von Heijne G (1998). Genome-wide analysis of integral membrane proteins from eubacterial, archaean, and eukaryotic organisms. Protein Sci.

[B2] Krogh A, Larsson B, von Heijne G, Sonnhammer E (2001). Predicting transmembrane protein topology with a hidden Markov model: application to complete genomes. J Mol Biol.

[B3] Drews J (2000). Drug discovery: a historical perspective. Science.

[B4] von Heijne G (2007). The membrane protein universe: what's out there and why bother?. J Intern Med.

[B5] Käll L, Krogh A, Sonnhammer E (2004). A combined transmembrane topology and signal peptide prediction method. J Mol Biol.

[B6] von Heijne G (1992). Membrane protein structure prediction. Hydrophobicity analysis and the positive-inside rule. J Mol Biol.

[B7] Sonnhammer E, von Heijne G, Krogh A (1998). A hidden Markov model for predicting transmembrane helices in protein sequences. Proc Int Conf Intell Syst Mol Biol.

[B8] Tusnády G, Simon I (1998). Principles governing amino acid composition of integral membrane proteins: application to topology prediction. J Mol Biol.

[B9] Rost B, Fariselli P, Casadio R (1996). Topology prediction for helical transmembrane proteins at 86% accuracy. Protein Sci.

[B10] Jones D (2007). Improving the accuracy of transmembrane protein topology prediction using evolutionary information. Bioinformatics.

[B11] Viklund H, Elofsson A (2004). Best alpha-helical transmembrane protein topology predictions are achieved using hidden Markov models and evolutionary information. Protein Sci.

[B12] Käll L, Krogh A, Sonnhammer E (2005). An HMM posterior decoder for sequence feature prediction that includes homology information. Bioinformatics.

[B13] Käll L, Sonnhammer E (2002). Reliability of transmembrane predictions in whole-genome data. FEBS Lett.

[B14] Melén K, Krogh A, von Heijne G (2003). Reliability measures for membrane protein topology prediction algorithms. J Mol Biol.

[B15] Martelli PL, Fariselli P, Casadio R (2003). An ENSEMBLE machine learning approach for the prediction of all-alpha membrane proteins. Bioinformatics.

[B16] Nilsson J, Persson B, von Heijne G (2000). Consensus predictions of membrane protein topology. FEBS Lett.

[B17] Ikeda M, Arai M, Lao DM, Shimizu T (2002). Transmembrane topology prediction methods: a re-assessment and improvement by a consensus method using a dataset of experimentally-characterized transmembrane topologies. In Silico Biol.

[B18] Taylor PD, Attwood TK, Flower DR (2003). BPROMPT: A consensus server for membrane protein prediction. Nucleic Acids Res.

[B19] Altschul S, Gish W, Miller W, Myers E, Lipman D (1990). Basic local alignment search tool. J Mol Biol.

[B20] Lassmann T, Sonnhammer E (2005). Kalign—an accurate and fast multiple sequence alignment algorithm. BMC Bioinformatics.

[B21] Altschul S, Madden T, Schäffer A, Zhang J, Zhang Z, Miller W, Lipman D (1997). Gapped BLAST and PSI-BLAST: a new generation of protein database search programs. Nucleic Acids Res.

[B22] Nielsen H, Krogh A (1998). Prediction of signal peptides and signal anchors by a hidden Markov model. Proc Int Conf Intell Syst Mol Biol.

[B23] Schölkopf B, Smola A (2002). Learning with Kernels Support Vector Machines.

[B24] Noble W (2006). What is a support vector machine?. Nat Biotechnol.

[B25] Arai M, Mitsuke H, Ikeda M, Xia J, Kikuchi T, Satake M, Shimizu T (2004). ConPred II: a consensus prediction method for obtaining transmembrane topology models with high reliability. Nucleic Acids Res.

[B26] Tusnády G, Kalmár L, Simon I (2008). TOPDB: topology data bank of transmembrane proteins. Nucleic Acids Res.

[B27] Chang CC, Lin CJ (2001). LIBSVM: a library for support vector machines.

[B28] Sonnhammer E, Wootton J (2001). Integrated graphical analysis of protein sequence features predicted from sequence composition. Proteins.

[B29] Dowell R, Jokerst R, Day A, Eddy S, Stein L (2001). The distributed annotation system. BMC Bioinformatics.

[B30] Messina DN, Sonnhammer EL (2009). DASher: a stand alone protein sequence client for DAS, the Distributed Annotation System. Bioinformatics.

